# A Case of Hæmoptysis

**Published:** 1888-03

**Authors:** Siegel Roush

**Affiliations:** Hillsboro, O.


### A Case of Hæmoptysis.

DEPENDENT ON A FOREIGN BODY IN THE LUNGS.

BY SIEGEL ROUSH, M. D., HILLSBORO, O.

About two months ago P. S., male, æt. 35 years, came to my office for a troublesome cough attended with occasional hemorrhage of the lungs. Patient was emaciated, weak, appetite poor, rapid and labored respiration, bowels alternating with constipation and diarrhœa, and, in short, presented all the symptoms of phthisis well advanced. The chest was well developed and physical examination revealed nothing abnormal except a few mucous rales in the region of the bifurcation of the trachea.

The family history was exceptionally good, there being no hereditary tendency to pulmonary disease whatever. The cough had been of nearly a year's standing, and failing to find any other cause for the trouble other than true pulmonary tuberculosis, I began treatment accordingly.

Under tonic treatment, a vegetable diet and improved hygiene, the patient became decidedly better and was, a few weeks ago dismissed, and he resumed his work—being that of a manual laborer.

A few days ago, however, he contracted a severe cold which again brought on paroxysms of coughing and, during one of these violent attacks, he expelled, together with a considerable amount of muco-purulent matter and blood, a molar tooth.

Upon inquiring I ascertained that about six years ago he received a blow on the side of the jaw which loosened one of his molar teeth from its socket, and, as he supposed, was swallowed. This information made the case a plain one and I did not hesitate to conclude that the cough and hemorrhage had been dependent upon the irritation and ulceration caused by the presence of this foreign body in the lungs.

Since the expulsion of the tooth, the cough and hemorrhage have almost wholly disappeared and I predict that the future phthisical symptoms in this case will be *nil.*

